# ACKNOWLEDGEMENTS TO REVIEWERS

**DOI:** 10.2174/1573403X1906231002102918

**Published:** 2023-10-02

Bentham Science Publishers would like to thank and appreciate the co-operation from all reviewers for their constructive comments and feedback on the manuscripts submitted to **“Current Cardiology Reviews”**. Their efforts have contributed greatly to the high quality and continuous growth of the journal. Given below is the list of reviewers who reviewed articles for the Journal during 2023:

Ahmed S. Abuzaid (USA)

Yuichiro Adachi (USA)

Kandasamy N. Aruljothi (India)

Sandra Arvidsson (Sweden)

Bela Balint (Serbia and Montenegro)

Jiali Bao (China)

Alessandro Bartoloni (Italy)

Federico J. Benetti (Argentina)

Sergio Berti (Italy)

Salima A. Bhimani (USA)

Kamalesh Chakravarty (India)

Melody Chemaly (Sweden)

Yang Chen (USA)

Wing K. Cheung (United Kingdom)

Erika A. Costa Cortez (Brazil)

Hehe Cui (China)

Nilsa R. Damaceno-Rodrigues Brazil)

Paulo M. Dourado (Brazil)

Mark W. Doyle (USA)

Nevzat Erdil (Turkey)

Jinqi Fan (China)

Monica Galli, (Italy)

Zainab Gandhi (USA)

Hui Gong (China)

Elena A. Grigoricheva (Russia)

Evgeny V. Grigoriev (Russia)

Karl H. Grinnemo (Sweden)

Adila A. Hamid (Malaysia)

Gerd Heusch (Germany)

Shamanna S. Iyengar (India)

Akhlesh K. Jain (India)

Manoj K. Jena (India)

Ageliki A. Karatza, Greece)

Wissam I. Khalife (USA)

Lloyd W. Klein (USA)

RICHARD kovacs (USA)

Filip Kucera (United Kingdom)

Manish Kumar (USA)

Likuo Kuo (Taiwan)

Martin Landsberger (Germany)

Linda Larsson Sweden)

Tae-soo Lee (South Korea)

Shaoxiong Li (China)

Wei Lin (China)

Zhen Liu (China)

Nani Maharani (Indonesia)

Duc Tho Mai (Japan)

JG Mill (Brazil)

Mritunjay K. Mishra (India)

Takashi Murashita (USA)

Francesco Nappi (France)

Kazumitsu Nawata (Japan)

Mark A.J. Newman (Australia)

Thach Nguyen (USA)

Masaaki Okutsu (Japan)

James Todd Pearson (Australia)

Soontaree Petchdee, (Thailand)

Claudio Picariello (Italy)

Priyia Pusparajah (Malaysia)

Lingbo Qian (China)

Vladimir Rafalskiy (Russia)

Hugo R. Ramos (Argentina)

Wan Shakira Rodzlan Hasani (Malaysia)

Mary N. Sheppard (United Kingdom)

Adam Sukiennik (Poland)

Taner Ulus (Turkey)

Gurpreet Singh Wander (India)

Tao Wang (China)

Mistiaen Wilhelm (Belgium)

Derek A William (USA)

Yang Yu (China)

Lingfang Zeng (United Kingdom)

Wenjing Zeng (China)

Li Zhang (China)

